# Fully automated deep learning powered calcium scoring in patients undergoing myocardial perfusion imaging

**DOI:** 10.1007/s12350-022-02940-7

**Published:** 2022-03-17

**Authors:** Thomas Sartoretti, Antonio G. Gennari, Elisabeth Sartoretti, Stephan Skawran, Alexander Maurer, Ronny R. Buechel, Michael Messerli

**Affiliations:** 1 Department of Nuclear Medicine, University Hospital Zurich / University of Zurich, Ramistrasse 100, 8091 Zurich, Switzerland; 2 University of Zurich, Zurich, Switzerland; 3 Maastricht University Medical Center, Maastricht University, Maastricht, the Netherlands

**Keywords:** CAD, Atherosclerosis, CT, SPECT, Diagnostic and prognostic application

## Abstract

**Background:**

To assess the accuracy of fully automated deep learning (DL) based coronary artery calcium scoring (CACS) from non-contrast computed tomography (CT) as acquired for attenuation correction (AC) of cardiac single-photon-emission computed tomography myocardial perfusion imaging (SPECT-MPI).

**Methods and Results:**

Patients were enrolled in this study as part of a larger prospective study (NCT03637231). In this study, 56 Patients who underwent cardiac SPECT-MPI due to suspected coronary artery disease (CAD) were prospectively enrolled. All patients underwent non-contrast CT for AC of SPECT-MPI twice. CACS was manually assessed (serving as standard of reference) on both CT datasets (*n* = 112) and by a cloud-based DL tool. The agreement in CAC scores and CAC score risk categories was quantified. For the 112 scans included in the analysis, interscore agreement between the CAC scores of the standard of reference and the DL tool was 0.986. The agreement in risk categories was 0.977 with a reclassification rate of 3.6%. Heart rate, image noise, body mass index (BMI), and scan did not significantly impact (*p*=0.09 - *p*=0.76) absolute percentage difference in CAC scores.

**Conclusion:**

A DL tool enables a fully automated and accurate estimation of CAC scores in patients undergoing non-contrast CT for AC of SPECT-MPI.

**Supplementary Information:**

The online version contains supplementary material available at 10.1007/s12350-022-02940-7.

## Introduction

Single-photon-emission computed tomography myocardial perfusion imaging (SPECT-MPI) is a well-established cardiac imaging modality that provides diagnostic and prognostic information in patients with known or suspected coronary artery disease (CAD).^1–5^ In SPECT-MPI, stress-induced perfusion abnormalities are visualized and quantified. Thus, the extent and severity of ischemia, scar burden and left ventricular systolic function and volume can be assessed.^6^

As SPECT-MPI is susceptible to soft-tissue attenuation, potentially compromising its diagnostic value,^1^ international guidelines recommend the use of an attenuation correction (AC) method. Non-contrast computed tomography is commonly used for AC as it provides further clinically relevant image information. Moreover, non-contrast CT enables the visualization of calcifications which can be leveraged to quantify coronary artery calcium (CAC).^6^ The latter is considered an important biomarker and independent predictor of cardiovascular mortality and all-cause mortality in patients with CAD. Importantly, patients’ risk is stratified according to categories, with CAC scores > 400, for example, implying substantial risk of cardiovascular disease events and mortality.^7–10^

However, the quantitative assessment of CAC (so-called calcium scoring, CACS) is a time-consuming and tedious manual task.^11–13^ Nevertheless, performing CACS in patients undergoing SPECT-MPI is clinically valuable, and as previous studies have shown, CACS provides additional complementary and prognostically relevant information to SPECT-MPI findings.^6,14,15^

With recent advances in the field of artificial intelligence for medical imaging,^11–13,16,17^ deep learning (DL) based CACS has become feasible. Such tools enable the automatic quantification of CAC, thus alleviating the need to perform CACS manually.^11–13,17^ Thus, a DL-based CACS approach may enable the extraction of potentially valuable data from non-contrast CT scans as performed for AC of SPECT-MPI without having to allocate time and effort to perform CACS manually.

Accordingly, we sought to test the feasibility of performing automated DL-based CACS on non-contrast CT scans for AC of myocardial SPECT-MPI. We hypothesized that a DL tool would provide accurate CACS results in a fully automated manner relative to manual measurements as the standard of reference.

## Materials and Methods

### Study Subjects

In this single-center, institutional review board-approved intra-individual comparative study, we identified and enrolled 56 patients (46 male (82%), age: 63 ± 9 years, body mass index (BMI): 28 ± 5 kg/m^2^) who underwent clinically indicated SPECT-MPI on a CZT technology-based camera and non-contrast CT based AC for the exclusion of CAD (Table 1). Some of the patients in the current investigation have been included in previous studies.^1,8^ All patients were part of a prospective study cohort in which two non-contrast CT scans for AC were acquired per patient (NCT03637231): The first scan was acquired prior to pharmacological heart rate control. The second scan was acquired after pharmacological heart rate control to achieve a target heart rate of below 65 beats per minute (BPM). For each patient, both scans were analyzed to quantify scan-rescan reproducibility and variability of CAC scores.

**Table 1:** Demographics of study patients (*n* = 56)

Number of patients	56
Male patients, *n* (%)	46 (82%)
Age, years	63 ± 9
Height, cm	174.6 ± 9.7
BMI, kg/m^2^	28 ± 5
Cardiovascular risk factors, n (%)	
Smoking	17 (30%)
Diabetes mellitus	6 (11%)
Hypertension	32 (57%)
Dyslipidemia	29 (52%)
Cardiac history, *n* (%)	
Previous MI	0 (0%)
Previous ICA	1 (0%)
Previous CABG	0 (0%)
Symptoms, *n* (%)	
Asymptomatic	12 (21%)
Typical angina pectoris	6 (11%)
Atypical chest pain	21 (37%)
Dyspnea	14 (25%)

Values given are mean±standard deviation or absolute numbers and percentages in brackets

*BMI* Body mass index; *CABG* coronary artery bypass grafting; *ICA* invasive coronary angiography; *MI* myocardial infarction

### Cardiac SPECT-MPI and CACS Imaging

All patients underwent a 1-day ^99m^Tc-tetrofosmin stress-rest myocardial perfusion imaging (MPI) protocol in accordance with current guidelines^18^. Exercise bicycle stress test was performed according to a modified Bruce protocol, and pharmacological stress was induced by intravenous regadenoson, adenosine, or dobutamine infusion. At peak stress (i.e., after 3 minutes of induced stress by adenosine or after reaching 85% of the patient’s predicted maximum heart rate), a weight-adjusted dose of ^99m^Tc-tetrofosmin was injected. Electrocardiogram (ECG)-gated stress images were acquired on a gamma camera with CZT-based detectors (Discovery 530 NMc, GE Healthcare, Milwaukee, WI, USA) 60-90 minutes after the isotope injection. Thereafter, a tracer dose three times the stress dose was administered at rest, followed by image acquisition with the same protocol as for stress. Patients with a BMI < 35 kg·m^−2^ received approximately 130 megabecquerel (MBq) for stress and 390 MBq for rest acquisition, whereas patients with a BMI ≥ 35 kg·m^−2^ received 180 MBq for stress and 540 MBq for rest acquisition.

Additionally, all patients underwent a non-contrast CT on a newest-generation 256-slice CT scanner (Revolution CT, GE Healthcare) for the creation of attenuation maps of the chest. All scans were performed in cranio-caudal direction during inspiratory breath-hold with prospective ECG-triggering as reported previously.^19^ In each patient, two separate scans (tube current 440.5 ± 96 milliampers and tube voltage 120 kilovolts) were acquired: The first one prior to pharmacological heart rate control and the second one after the application of metoprolol to decrease heart rate below 65 BPM. For each acquisition, the following scan parameters were chosen: collimation of 256 ×  0.625 mm, *z*-coverage of 12-16 cm, field of view of 25 cm, and gantry rotation time of 280 milliseconds. As recommended elsewhere, images were reconstructed using filtered back projection (FBP).^20,21^ Effective radiation dose as quantified by the dose length product (DLP) multiplied with a conversion factor (0.014 mSv × mGy^−1^ × cm^−1^)^22^ was 0.6 ± 0.3 mSv.

### Manual Calcium Scoring

Images were transferred to a dedicated workstation (Advantage AW 4.4, GE Healthcare) with a dedicated CACS software (SmartScore 4.0, GE Healthcare). All pixels with an attenuation equal or above the lowest threshold (e.g., ≥ 130 HU for 120-kV scans) having an area ≥ 1 mm^2^ are automatically color marked, and lesions are manually selected by creating a region of interest around all lesions found in a coronary artery. The software then calculates the CAC score, as previously described.^20^ In brief, a score for each region of interest is calculated by multiplying the density score (i.e., the thresholds) and the area of calcifications. A total CAC score is then determined by adding up the scores for each CT slice. Importantly, the software computes an overall CAC score and vessel-wise CAC scores. Of note, the thresholds for CACS are only applied to pixels with a density equal or larger than the lowest threshold and an area of ≥ 1 mm^2^. This eliminates single pixels with a density above the thresholds due to noise. All datasets were analyzed by two experienced readers in random order, and measurements from both readers were averaged.^8^ CAC risk categories were defined according to the following CAC score boundaries: 0, 1-100, 101-400, > 400.

### Deep Learning Calcium Scoring

CAC scoring was performed by a fully automated DL-based CAC scoring tool (AVIEW CAC, Coreline Soft, access via https://cloud.corelinesoft.eu/login). In brief, the tool was trained on a 3-dimensional U-net architecture using non-enhanced cardiac CT scans acquired from multiple vendors and scanners as input data. No specific training data were included in this current study. A detailed description of this DL tool can be found elsewhere.^11,23^ In short, the network makes predictions with patches, and the segmentation mask is reconstructed with output patches. After reconstruction, only the coronary artery areas (i.e., left main coronary artery [LM], left anterior descending coronary artery [LAD], left circumflex coronary artery [LCX], and right coronary artery [RCA]) near the heart are filtered using the predicted Ventricle area. A multi-branch network is used consisting of the same features for coronary arteries and other coronary structures. It is based on U-Net (see also https://arxiv.org/abs/1505.04597) with residual units (see also https://arxiv.org/abs/1603.05027) in the feature extractors, and the input layers have shape 64 × 64 × 64 × 1 (3D patch with single channel). The output layers of two networks have shape 64 × 64 × 64 × channels. One branch has the last layer with 5 channels (with background) for coronary arteries, the other has the last layer with 8 channels (with background) for other coronary structures.

### Image Analysis

To quantify image noise, one reader (A.G.G., with 7 years of experience in cardio-thoracic imaging) placed region of interests (ROI) on three consecutive CT image slices in the left ventricle. The standard deviation of values in each ROI was considered as image noise. The values from the three measurements were averaged and were then considered representative for further analyses.

### Statistical Analysis

Descriptive statistics including mean ± standard deviation, counts, and percentages were used to present the results. To quantify the agreement of CAC scores, linear regression models were fitted, Bland-Altman analysis was performed, and two-way intraclass correlation coefficients (ICC) were computed. To quantify the agreement of CAC risk categories, weighted Kappa analysis was performed. The following scale was used to classify correlation coefficients: values of less than 0.20 were indicative of poor agreement; 0.21-0.40, fair agreement; 0.41-0.60, moderate agreement; 0.61-0.80, good agreement; and 0.81-1.00, excellent agreement. To compare values stratified by groups (such as CAC scores or heart rate), paired t-tests or wilcoxon signed-rank tests were used. Potential differences in reclassification rates of CAC score categories, paired *z*-tests of proportions were used. Lastly, a generalized linear model (GLM) using iteratively reweighted least squares to find the maximum likelihood estimates was fitted. The absolute percentage difference in CAC scores between the standard of reference and the DL tool was implemented as dependent variable and heart rate, image noise, BMI, and scan were considered as predictors. Two-tailed *p*-values < 0.05 were considered significant. All statistical analyses were performed in the R programming language (version 4.0.2; R Foundation for Statistical Computing, Vienna, Austria, https://www.R-project.org).

## Results

### Overall Accuracy of DL-CACS

**Figure 1:**
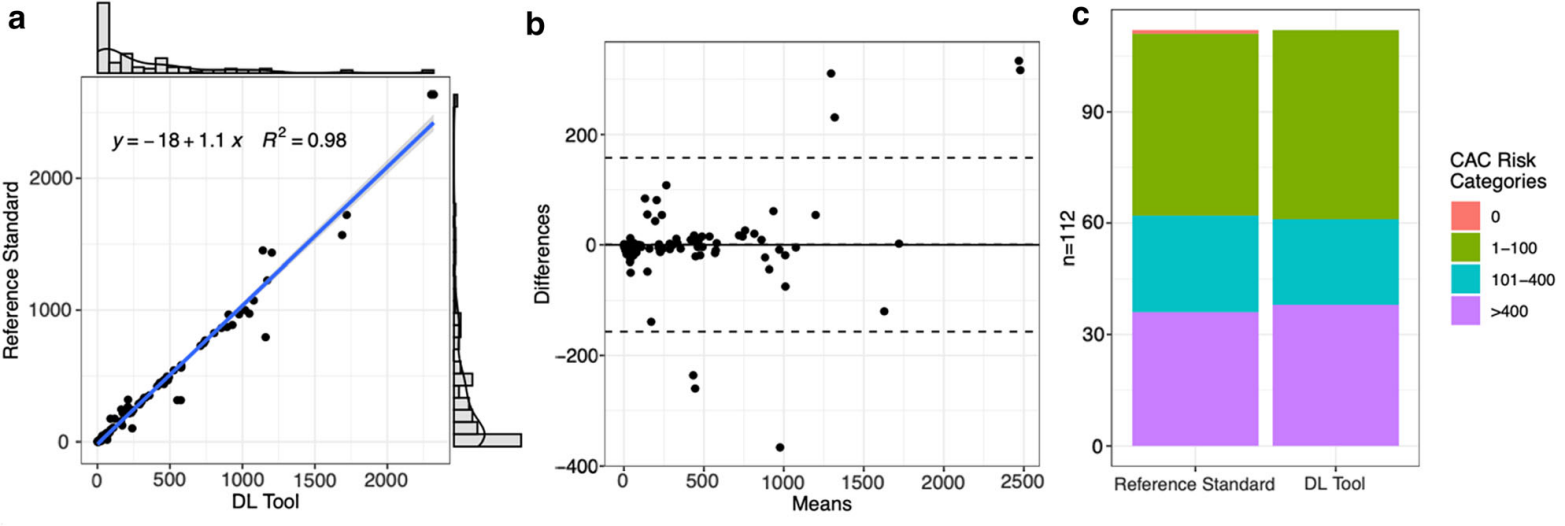
Visual representation of the study data including the data of all scans (*n* = 112): Linear regression model (**A**), Bland-Altman analysis (**B**), as well as CAC scores stratified by risk categories (**C**) of the manual coronary artery calcium scoring (i.e., standard of reference) and fully automated DL-CACS

A visual representation of the study results is provided in Figure 1. An overview of the data is provided in Table 2. For the overall analysis, each scan was considered for statistical analysis. Thus, 112 scans from 56 patients were considered. The DL tool successfully managed to automatically perform CAC scoring in all cases within 63 ± 48 s. The overall CAC score was 352.6 ± 491 for the standard of reference and 352.1 ± 461 for the DL tool. The regression model using CAC scores of the standard of reference as dependent variable and the CAC scores of the DL tool as predictor (*R*^2^ = 0.98, *p* < 0.001), exhibited a slope of 1.1 and an intercept of -18. Bland-Altman analysis (Fig. 1) revealed a bias of 0.44, a lower limit of agreement of − 156.8, and an upper limit of agreement of 157.7. Interscore agreement (ICC) between the CAC scores of the standard of reference and the DL tool was 0.986 (95%CI 0.979, 0.99).

**Table 2:** Confusion matrices of risk categories between DL-CACS and manual CACS. Weighted kappa value was 0.977

Manual CACS	DL-CACS				Total^a^	Underestimation^b^	Overestimation^b^	Concordance^b^
	0	1-100	101-400	> 400				
0	0	1	0	0	1	0 (0%)	1 (100%)	0 (0%)
1-100	0	49	0	0	49	0 (0%)	0 (0%)	49 (100%)
101-400	0	1	23	2	26	1 (3.8%)	2 (7.7%)	23 (88.5%)
>400	0	0	0	36	36	0 (0%)	0 (0%)	36 (100%)

CACS coronary artery calcium scoring; DL deep learning

^a^*n* = 112 scans from 56 patients

^b^ i.e., manual coronary artery calcium scoring as standard of reference

In terms of CAC risk categories, a reclassification rate of 3.6% (4 of 112 cases) was found between the standard of reference and the DL tool. In all 4 cases, reclassification was only by one category. Specifically, the DL tool falsely increased the class by one category in 3 cases and falsely decreased the class by one category in 1 case. Interclass agreement was 0.977 as determined by weighted Kappa analysis. Reasons for CAC score reclassification (i.e., under- or overestimation) of the DL tool in the 4 cases are provided in Table 3.

**Table 3:** Cases (4/112) of patients where DL-CACS led to reclassification of coronary risk

N	Age	Manual-CACS^a^	DL-CACS	DL-Classification	Reason for wrong classification of DL tool
1	75	174	90	Underestimated	Missed calcification in LCX
2	66	315	551	Overestimated	Aortic valve calcification falsely rated as CAC
3	66	315	575	Overestimated	Aortic valve calcification falsely rated as CAC
4	55	0	3	Overestimated	Image noise falsely rated as CAC

CACS coronary artery calcium scoring; DL deep learning; LCX left circumflex artery

i.e., manual coronary artery calcium scoring as standard of reference

The generalized model with heart rate, image noise, BMI, and scan (i.e., scan 1 or 2) as predictors revealed no significant impact of all variables (*p* = 0.09 – *p* = 0.76) on absolute percentage difference in CAC scores between the DL tool and the standard of reference.

### Per Vessel Analysis of DL-CACS

On a per-vessel basis, interscore agreement (ICC) between the standard of reference and the DL tool was 0.635 (95%CI 0.511, 0.734), 0.95 (95%CI 0.928, 0.965), 0.928 (95%CI 0.897, 0.95), and 0.991 (95%CI 0.988, 0.994) for LM, LAD, LCX, and RCA, respectively. Specifically, the CAC scores (standard of reference / DL tool) were 13.9 ± 32.1 / 29.9 ± 50.2 for LM, 174 ± 260.7 / 152.6 ± 212.2 for LAD, 54.5 ± 101.3 / 53.2 ± 106.2 for LCX, and finally 110.1 ± 194.6 / 116.4 ± 198.6 for RCA.

### Scan-Rescan Reproducibility and Variability

Heart rate was 65.7 ± 13.4 BPM for the first scan and 58.9 ± 6.4 BPM for the second scan (*p* < 0.001). An average CAC score variability of 6.7 ± 64.7 and 13.6 ± 57.6 between both scans was observed for the standard of reference and the DL tool, respectively, without significant differences between both methods (*p* = 0.45). Interscore agreement between both scans as quantified by ICC was 0.991 (95%CI 0.985, 0.995) and 0.992 (95%CI 0.987, 0.995) for the standard of reference and the DL tool, respectively.

In terms of quantification accuracy of the DL tool relative to manual measurements as the standard of reference, ICC of CAC scores was 0.984 (95%CI 0.973, 0.991) for the first scan and 0.988 (95%CI 0.979, 0.993) for the second scan. Reclassification rate for CAC risk categories was 1.8% for the first scan and 5.4% for the second scan (*p* < 0.001). Interclass agreement of CAC risk categories was 0.988 and 0.966 for the first and second scan, respectively, as determined by weighted Kappa analysis.

**Figure 2:**
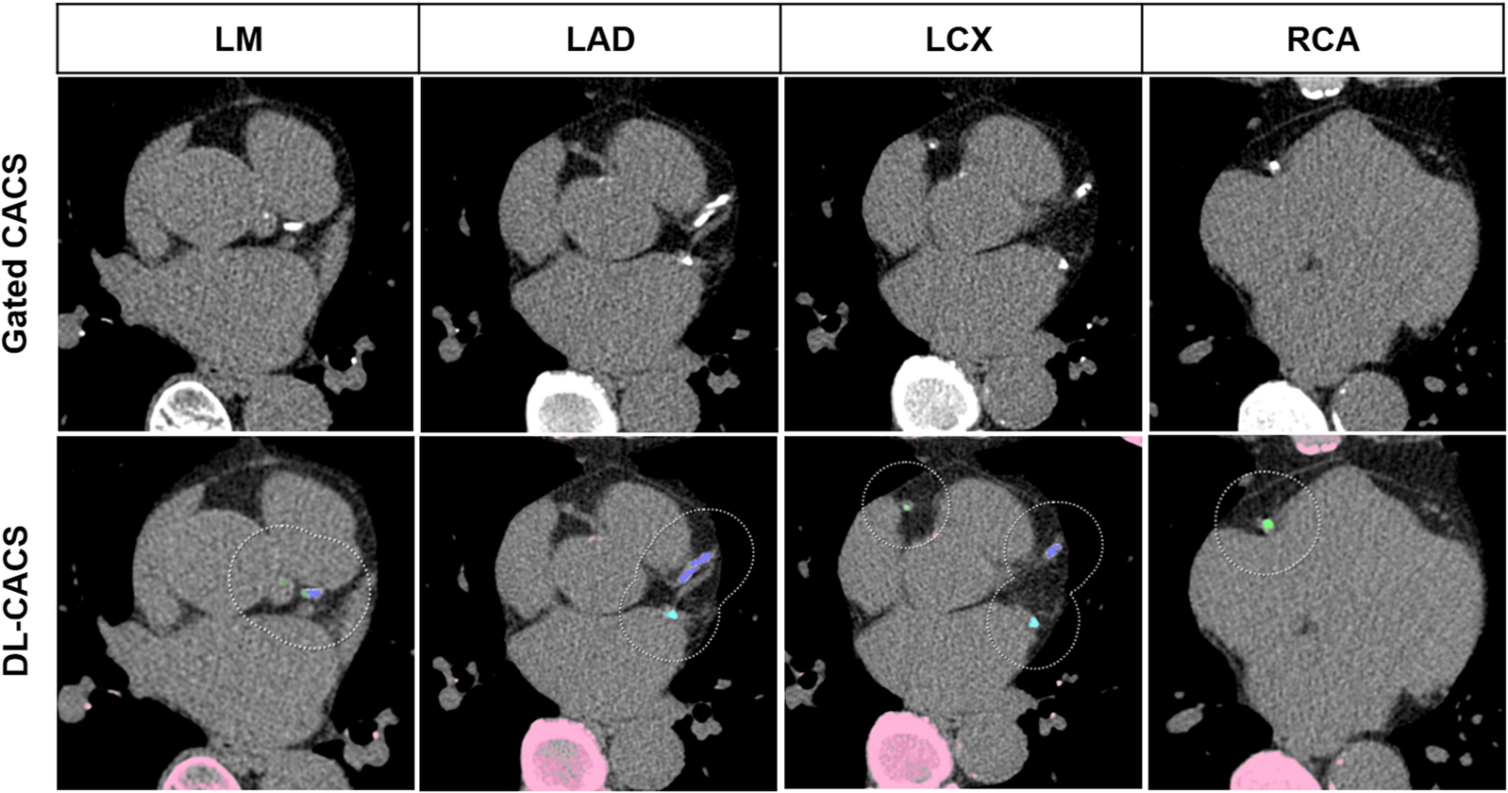
Representative CT images of a 64-year-old woman with a body mass index of 22.9 kg/m^2^ with severe coronary artery calcifications. Images from gated non-contrast CT scanner for the creation of attenuation maps is presented in the upper row and fully automated deep learning coronary artery calcium scoring (DL-CACS) is presented in lower row. Coronary calcifications in the left main (LM), left anterior descending (LAD), left circumflex artery (LCX), and right coronary artery (RCA), were correctly marked by the DL-CACS tool resulting in a total score of 894. The score from manual readout of CAC scan was 871

A representative case of the DL-CACS tool correctly identifying the coronary calcium burden in a patient is presented in Fig. 2.

## Discussion

In this single-center, prospective, intra-individual comparative study, we compared the quantitative accuracy of CACS as performed fully automatically on non-contrast CT acquired for AC of SPECT-MPI by a DL tool relative to manual measurements as the standard of reference.

Our data indicate that the accuracy of the DL tool is very high, with interscore agreement of CAC scores and interclass agreement of CAC risk categories being excellent as well. Furthermore, the scan-rescan evaluation confirmed quantification stability and robustness of the DL tool on par with that of manual measurements. Further, quantitative accuracy was not significantly influenced by heart rate, image noise or BMI, thus confirming the overall validity and value of the DL tool. Given these results, our study suggests that a DL tool may derive accurate CAC scores from non-contrast CT scans as acquired for AC of SPECT-MPI in a fully automated manner.

The use of AI-based solutions for automated CACS has only gained traction recently. Notably, Van Velzen et al. developed a DL-backed CACS approach that achieved ICC scores of as high as 0.99 and risk category reclassification rates of as low as 3% when using calcium scans from CCTA as input data.^12^ Vonder et al. showed that a DL-CACS tool achieved an ICC score of 0.96 and a risk category reclassification rate of 2% relative to manual measurements in a cohort of 997 patients who underwent calcium scans of CCTA.^11^ To the best of our knowledge, this is the first study systematically assessing the validity of a DL approach for CACS on CT data as acquired for SPECT-MPI. Here, we achieved a similarly high performance as reported in previous studies with an ICC score as high as 0.986 and risk category reclassification rates of 3.6% between the DL tool and manual measurements.

Notably, while the overall agreement of CAC scores was very high (ICC: 0.986), we observed differences between the various coronary arteries. Specifically, for the LM artery, the interscore agreement of CAC scores was lower (ICC: 0.635) than that of the other coronary arteries (ICC ranging from 0.928 to 0.991). On average, the LM artery exhibited less CAC than the other vessels. Even small deviations in CAC scores between the reference standard and the DL tool may thus have caused considerable reductions in the the ICC. Furthermore, it should ne noted that the DL tools allocation of CAC to the various vessels may have differed slightly from that of the expert readers. Thus, in some cases the DL tool may have accurately recognized CAC but may have falsely allocated it to a different vessel which would then have resulted in lower ICC values for a given vessel.

On another note, it should be mentioned that our reclassification rates were slightly higher than those of previous studies. The reason is, firstly, that at least compared to Vonder et al. we had more and narrower risk categories.^11^ Secondly, our data correspond to a real validation set. That is, the DL tool was not retrained and optimized with data from our study cohort and institution. In contrast, Van Velzen et al. implemented additional protocol-specific training to further optimize the performance of their DL tool.^12^ Furthermore, it should be noted that the study cohort may have differed between ours and previous studies. In our study, nearly all patients presented with CAC as our patients all had a moderate to high pre-test probability of CAD, as can be expected form patients referred for SPECT-MPI. In contrast, in patients undergoing CCTA, the pre-test probability of CAD is lower. Notably, Vonder et al. used patient data from a population-based cardiovascular screening trial.^11^ In such a cohort, the proportion of patients with very little to no calcium is probably much higher than in our cohort.

Lastly, we would like to highlight the value of CACS in patients undergoing SPECT-MPI. Chang et al. followed up a cohort of 1126 generally asymptomatic patients without previous CAD who had undergone SPECT-MPI and CACS within a close period of time. The authors concluded that at a median follow-up of 6.9 years, CACS and SPECT-MPI results provided both independent and complementary prognostic information for total cardiac events and all-cause death/myocardial infarction.^14^ Furthermore, Engbers et al. showed that in a cohort of 4897 symptomatic patients, CACS and SPECT-MPI findings were independent predictors of major adverse cardiac events (MACE). The authors thus recommended routinely performing CACS in adjunct to SPECT-MPI in patients suspected of CAD in an effort to further improve risk prediction during follow-up.^15^

In addition, it should be mentioned that besides the prognostic and diagnostic value of CACS, it is also otherwise useful to extract the maximum information from the data acquired as part of an examination. Especially when considering that a SPECT-MPI examination involves radiation exposure, one should strive to maximize the information content and diagnostic value of the examination.^24^ Due to the fact that DL-based CACS does not require any further time investment, the use of such a tool is particularly useful and desirable in this context. Importantly, it should be considered that the results of this study carry implications for a wide range of diagnostic imaging modalities in nuclear cardiology. Specifically, the DL tool presented in this study may also be deployed for other imaging modalities such as hybrid cardiac PET/CT examinations.

Our study has the following limitations: First, this was a single-center study with a limited number of subjects. Secondly, all images were acquired on a single scanner with a single imaging protocol. We acknowledge that varying scan parameters may influence the results. Thirdly, we acknowledge that quantification accuracy may depend on the study cohort examined. Future studies should assess the quantification accuracy of the DL tool in patients with no or very high CAC.

## New Knowledge Gained

A deep learning powered approach can fully automatically and accurately perform coronary artery calcium scoring from non-contrast computed tomography images as acquired for attenuation correction of single-photon-emission computed tomography myocardial perfusion imaging. This approach allows for the opportunistic and effortless extraction of coronary artery calcium scores in patients undergoing single-photon-emission computed tomography myocardial perfusion imaging.

## Conclusion

Our study shows that a DL tool enables a fully automated and accurate estimation of CAC scores in patients undergoing SPECT-MPI. Thus, DL-based CACS may facilitate the further implementation of CAC scores as a routine imaging marker determined during the workup of SPECT-MPI examinations.

## Supplementary Information

Below is the link to the electronic supplementary material.

Supplementary file1 (PPTX 349 kb)

Supplementary file2 (PPTX 446 kb)

## Abbreviations

SPECT-MPI: Single-photon-emission computed tomography myocardial perfusion imaging
CAD: Coronary artery disease
AC: Attenuation correction
CAC: Coronary artery calcium
CACS: Coronary artery calcium scoring
DL: Deep learning
BMI: Body mass index
BPM: Beats per minute
ROI: Region of interest
ICC: Intraclass correlation coefficient
GLM: Generalized linear model
LM: Left main coronary artery
LAD: Left anterior descending coronary artery
LCX: Left circumflex coronary artery
RCA: Right coronary artery
CCTA: Coronary computed tomography angiography
MACE: Major adverse cardiac events
