# Supplementary material for: Fully automated deep learning powered calcium scoring in patients undergoing myocardial perfusion imaging
Source: J Nucl Cardiol. 2022 Mar 17;30(1):313–20. doi: 10.1007/s12350-022-02940-7 (PMC9984313; doi:10.1007/s12350-022-02940-7)
Supplement: Supplementary file 1 — Supplementary file1 (PPTX 349 kb) [file 12350_2022_2940_MOESM1_ESM.pptx]

## Slide 1
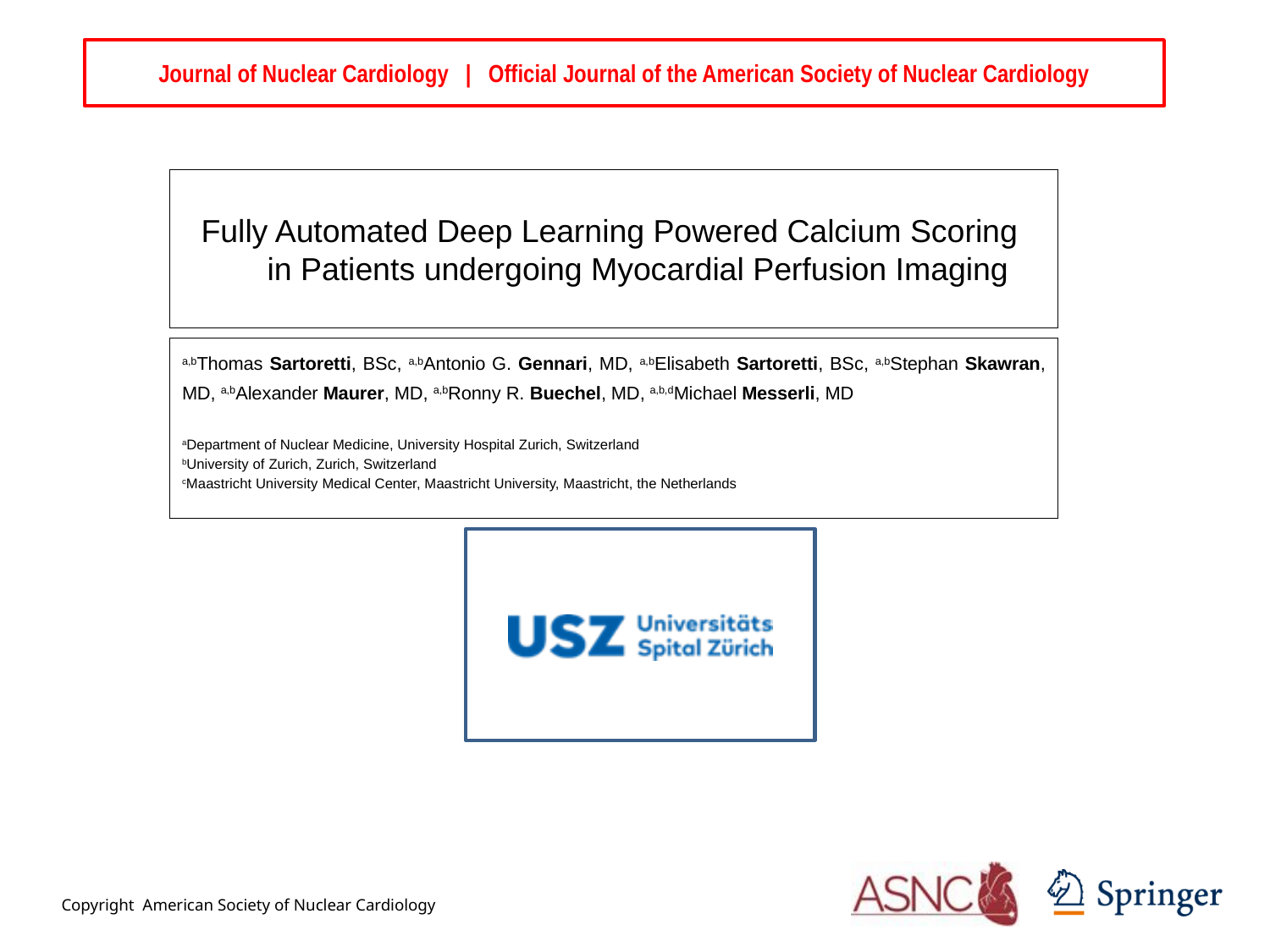

Journal of Nuclear Cardiology | Official Journal of the American Society of Nuclear Cardiology
# Fully Automated Deep Learning Powered Calcium Scoring in Patients undergoing Myocardial Perfusion Imaging
a,bThomas Sartoretti, BSc, a,bAntonio G. Gennari, MD, a,bElisabeth Sartoretti, BSc, a,bStephan Skawran, MD, a,bAlexander Maurer, MD, a,bRonny R. Buechel, MD, a,b,dMichael Messerli, MD
aDepartment of Nuclear Medicine, University Hospital Zurich, Switzerland
bUniversity of Zurich, Zurich, Switzerland
cMaastricht University Medical Center, Maastricht University, Maastricht, the Netherlands
Copyright American Society of Nuclear Cardiology

## Slide 2
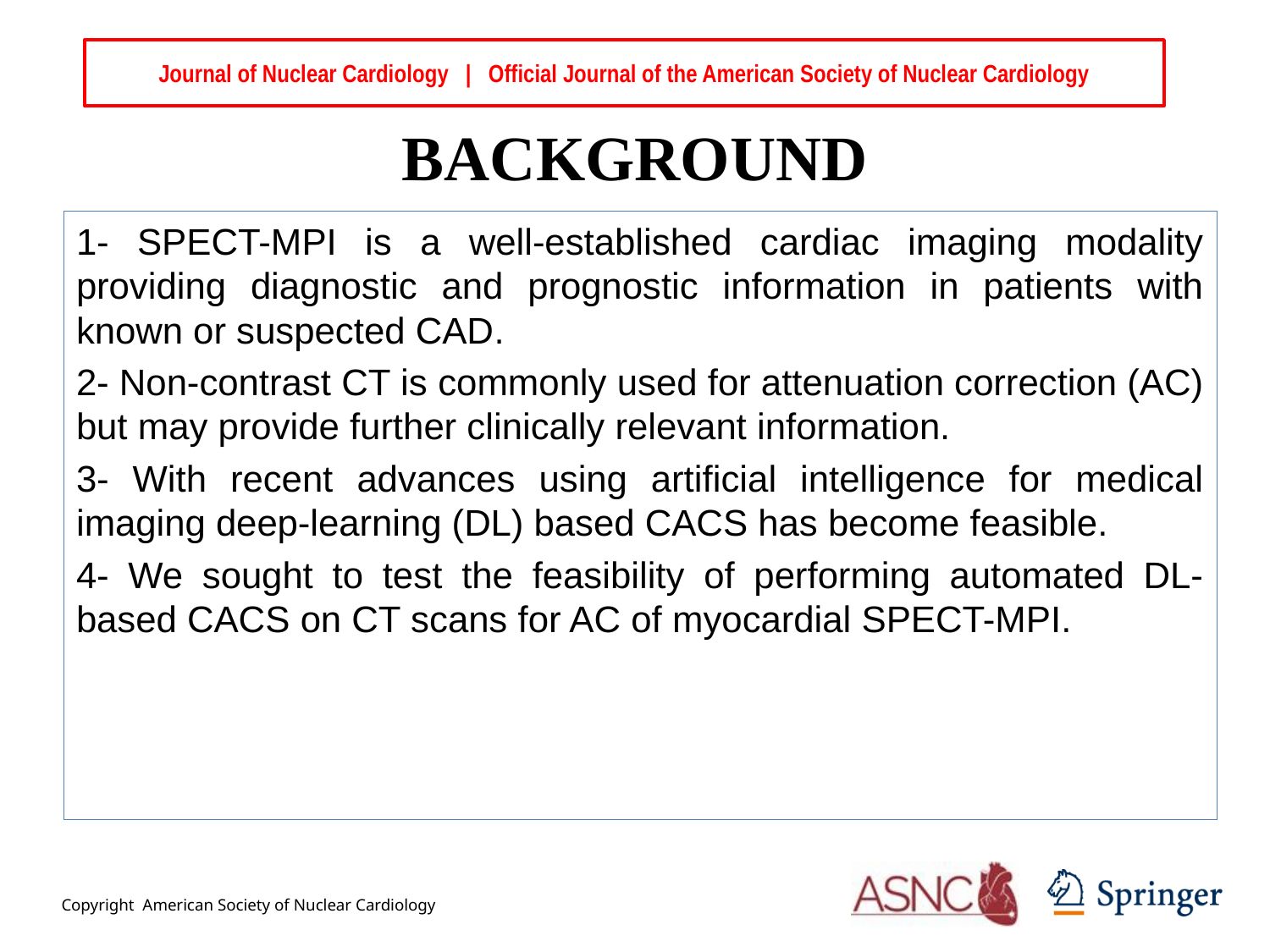

Journal of Nuclear Cardiology | Official Journal of the American Society of Nuclear Cardiology
# BACKGROUND
1- SPECT-MPI is a well-established cardiac imaging modality providing diagnostic and prognostic information in patients with known or suspected CAD.
2- Non-contrast CT is commonly used for attenuation correction (AC) but may provide further clinically relevant information.
3- With recent advances using artificial intelligence for medical imaging deep-learning (DL) based CACS has become feasible.
4- We sought to test the feasibility of performing automated DL-based CACS on CT scans for AC of myocardial SPECT-MPI.
Copyright American Society of Nuclear Cardiology

## Slide 3
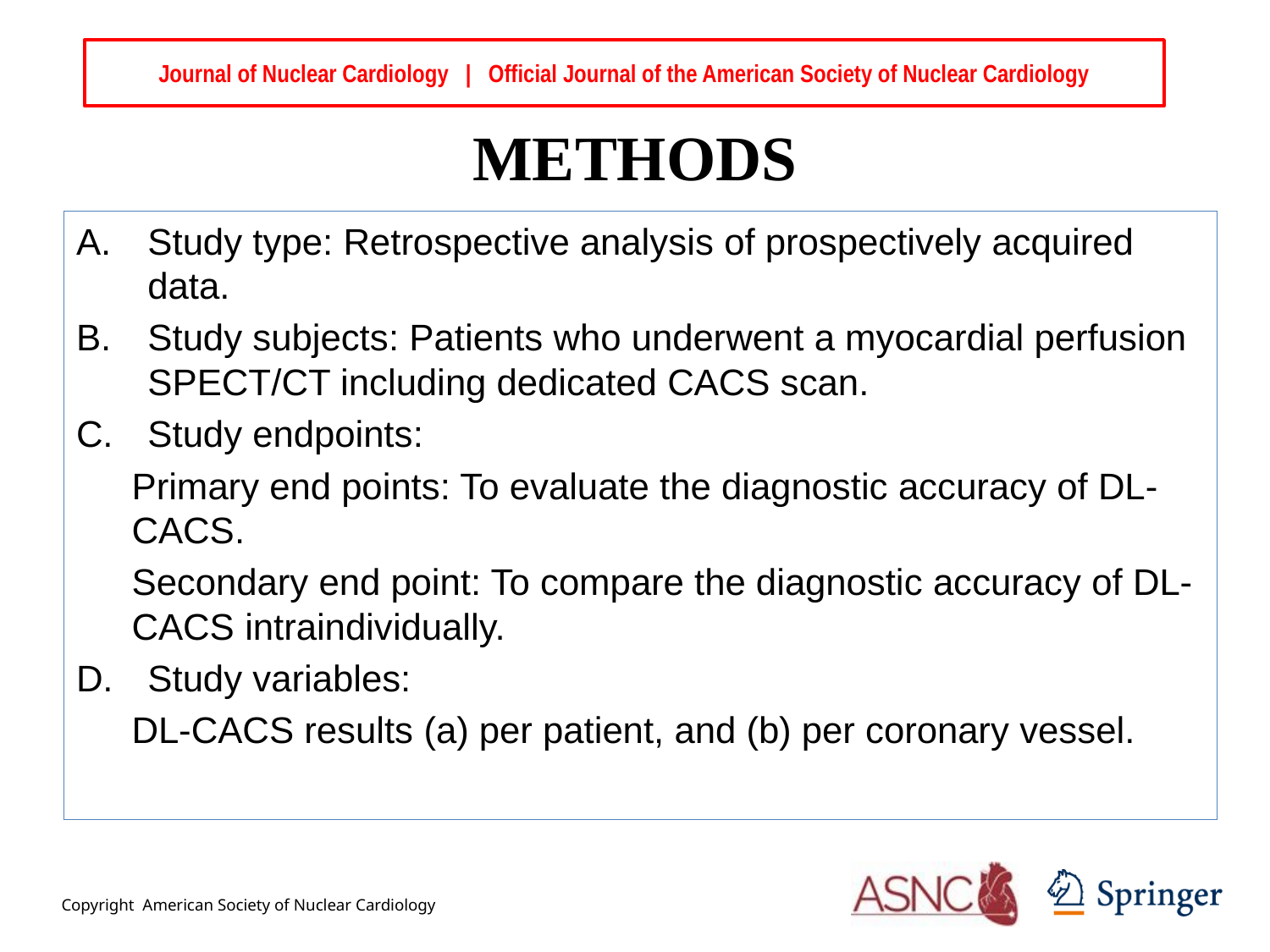

Journal of Nuclear Cardiology | Official Journal of the American Society of Nuclear Cardiology
# METHODS
Study type: Retrospective analysis of prospectively acquired data.
Study subjects: Patients who underwent a myocardial perfusion SPECT/CT including dedicated CACS scan.
Study endpoints:
Primary end points: To evaluate the diagnostic accuracy of DL-CACS.
Secondary end point: To compare the diagnostic accuracy of DL-CACS intraindividually.
Study variables:
DL-CACS results (a) per patient, and (b) per coronary vessel.
Copyright American Society of Nuclear Cardiology

## Slide 4
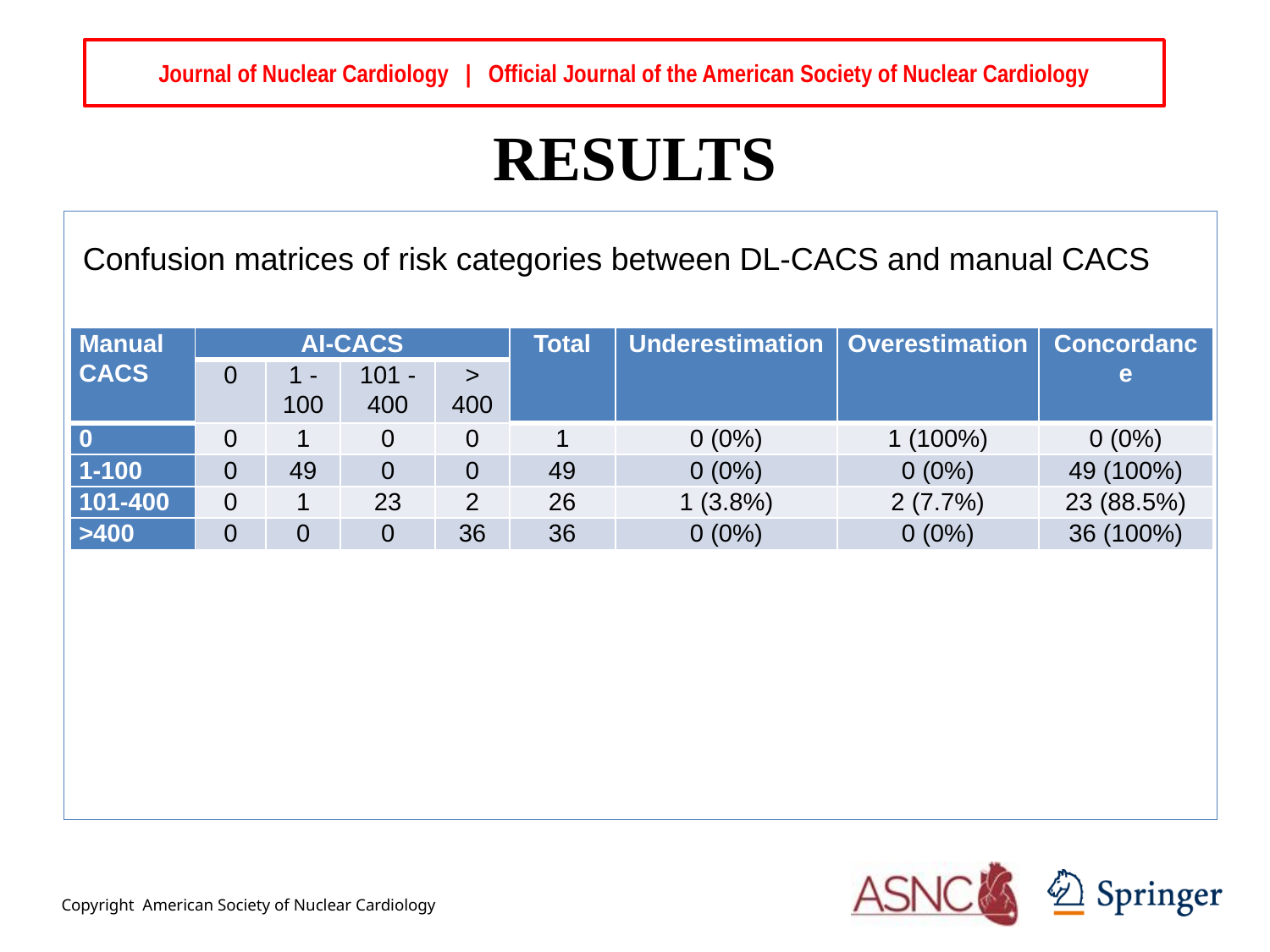

Journal of Nuclear Cardiology | Official Journal of the American Society of Nuclear Cardiology
# RESULTS
Confusion matrices of risk categories between DL-CACS and manual CACS
| Manual CACS | AI-CACS | | | | Total | Underestimation | Overestimation | Concordance |
| --- | --- | --- | --- | --- | --- | --- | --- | --- |
| | 0 | 1 - 100 | 101 - 400 | > 400 | | | | |
| 0 | 0 | 1 | 0 | 0 | 1 | 0 (0%) | 1 (100%) | 0 (0%) |
| 1-100 | 0 | 49 | 0 | 0 | 49 | 0 (0%) | 0 (0%) | 49 (100%) |
| 101-400 | 0 | 1 | 23 | 2 | 26 | 1 (3.8%) | 2 (7.7%) | 23 (88.5%) |
| >400 | 0 | 0 | 0 | 36 | 36 | 0 (0%) | 0 (0%) | 36 (100%) |
Copyright American Society of Nuclear Cardiology

## Slide 5
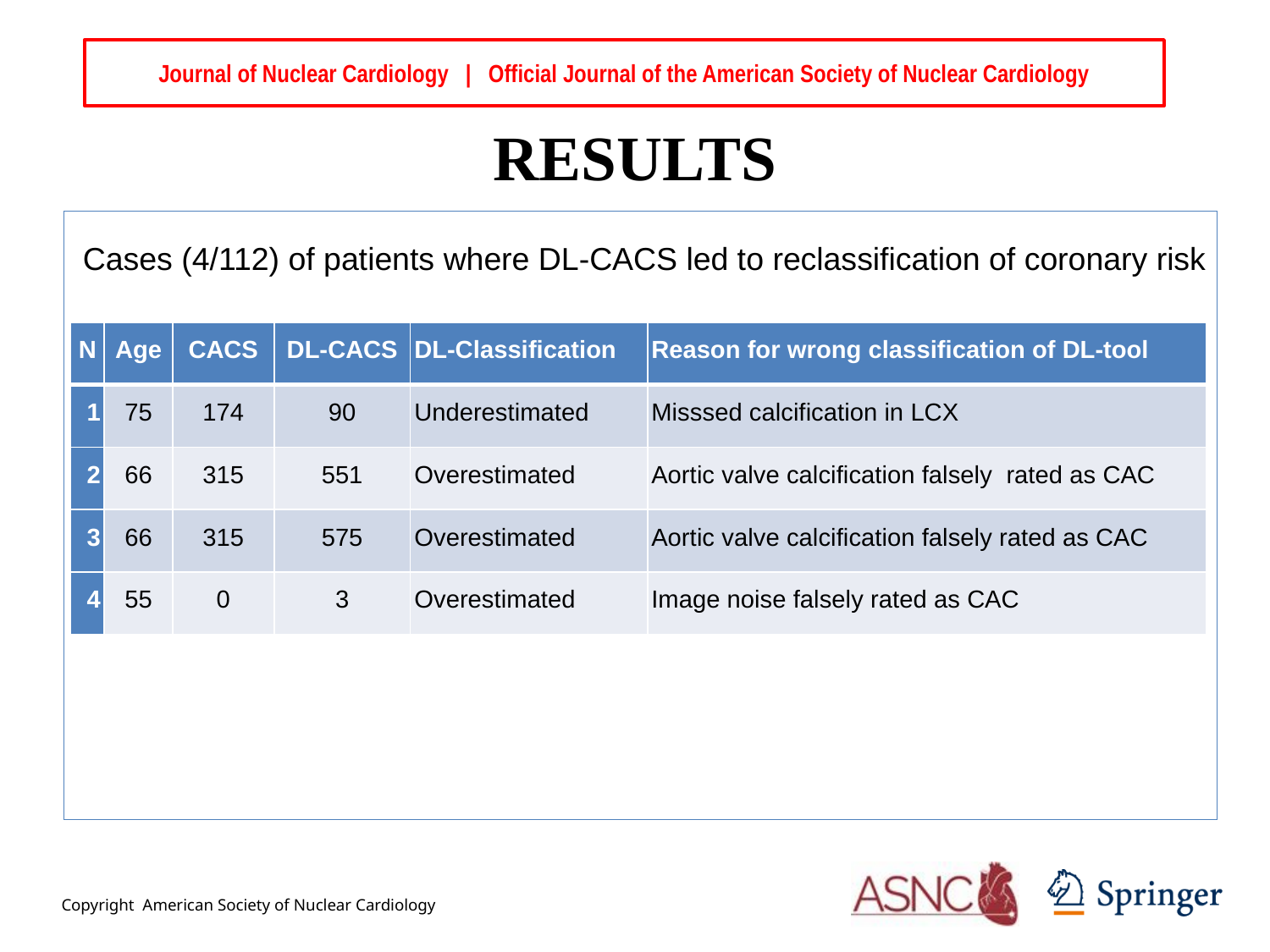

Journal of Nuclear Cardiology | Official Journal of the American Society of Nuclear Cardiology
# RESULTS
Cases (4/112) of patients where DL-CACS led to reclassification of coronary risk
| N | Age | CACS | DL-CACS | DL-Classification | Reason for wrong classification of DL-tool |
| --- | --- | --- | --- | --- | --- |
| 1 | 75 | 174 | 90 | Underestimated | Misssed calcification in LCX |
| 2 | 66 | 315 | 551 | Overestimated | Aortic valve calcification falsely rated as CAC |
| 3 | 66 | 315 | 575 | Overestimated | Aortic valve calcification falsely rated as CAC |
| 4 | 55 | 0 | 3 | Overestimated | Image noise falsely rated as CAC |
Copyright American Society of Nuclear Cardiology

## Slide 6
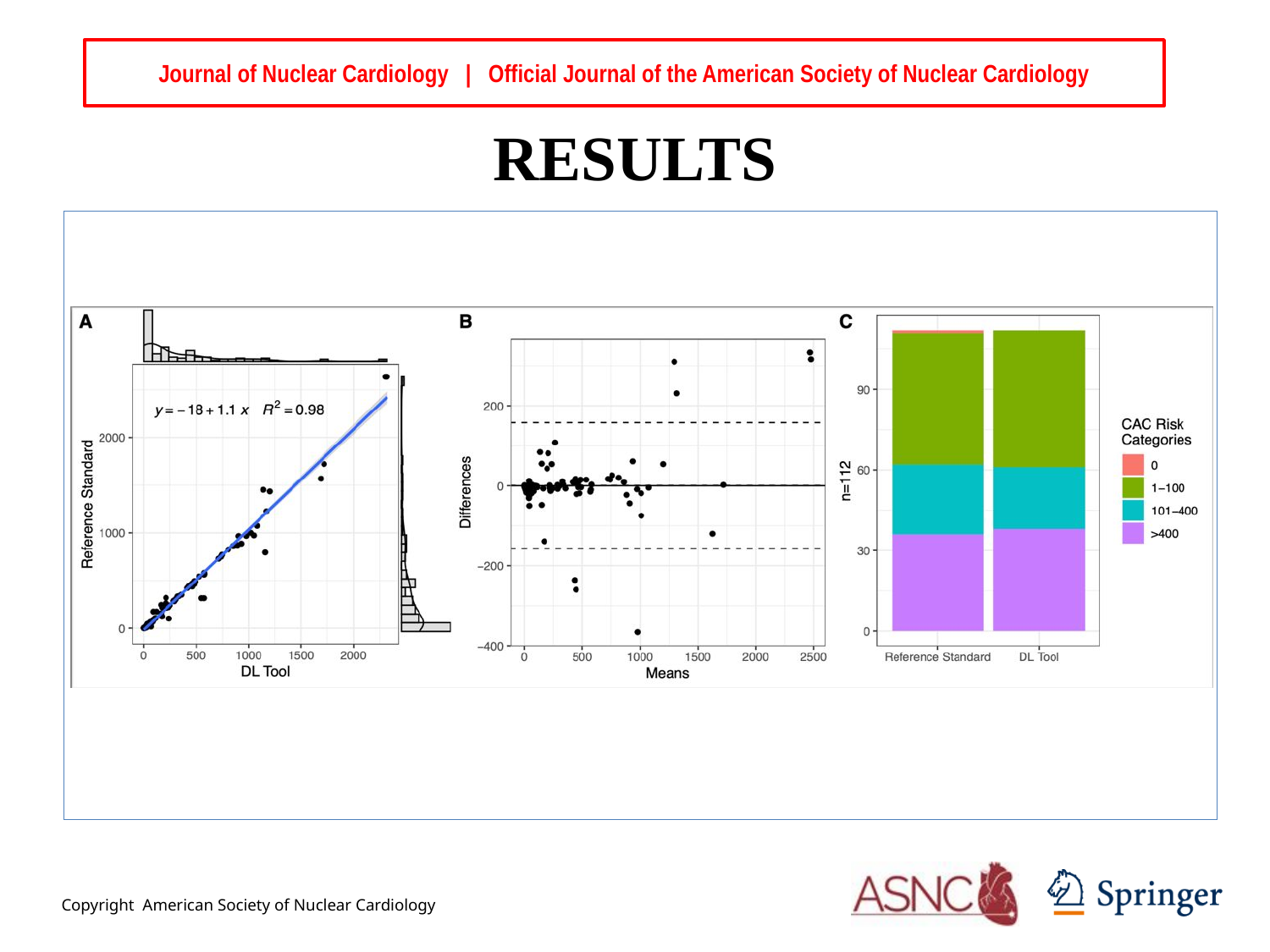

Journal of Nuclear Cardiology | Official Journal of the American Society of Nuclear Cardiology
# RESULTS
Copyright American Society of Nuclear Cardiology

## Slide 7
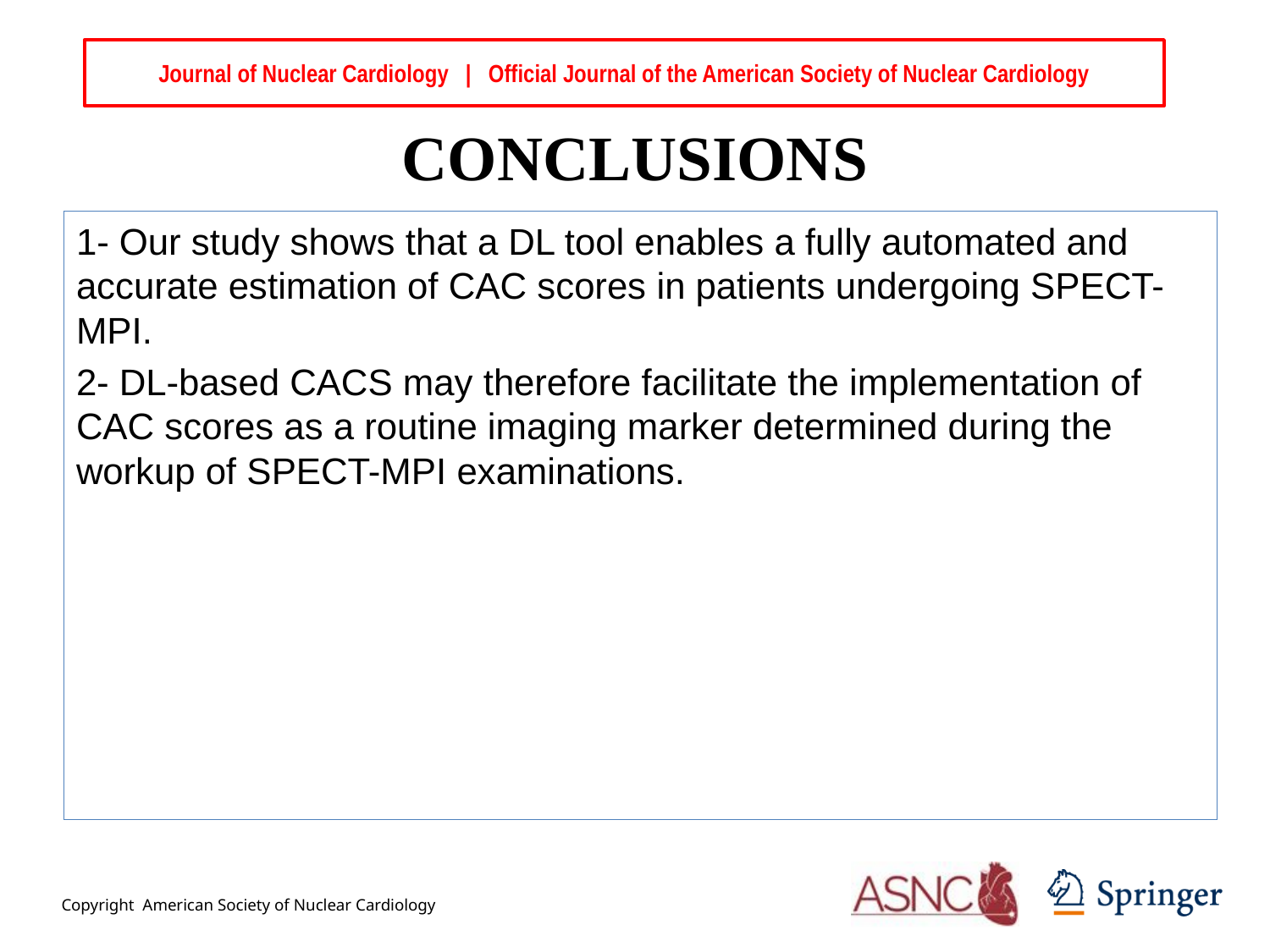

Journal of Nuclear Cardiology | Official Journal of the American Society of Nuclear Cardiology
# CONCLUSIONS
1- Our study shows that a DL tool enables a fully automated and accurate estimation of CAC scores in patients undergoing SPECT-MPI.
2- DL-based CACS may therefore facilitate the implementation of CAC scores as a routine imaging marker determined during the workup of SPECT-MPI examinations.
Copyright American Society of Nuclear Cardiology
