# Supplementary material for: Fully automated deep learning powered calcium scoring in patients undergoing myocardial perfusion imaging
Source: J Nucl Cardiol. 2022 Mar 17;30(1):313–20. doi: 10.1007/s12350-022-02940-7 (PMC9984313; doi:10.1007/s12350-022-02940-7)
Supplement: Supplementary file 2 — Supplementary file2 (PPTX 446 kb) [file 12350_2022_2940_MOESM2_ESM.pptx]

## Slide 1
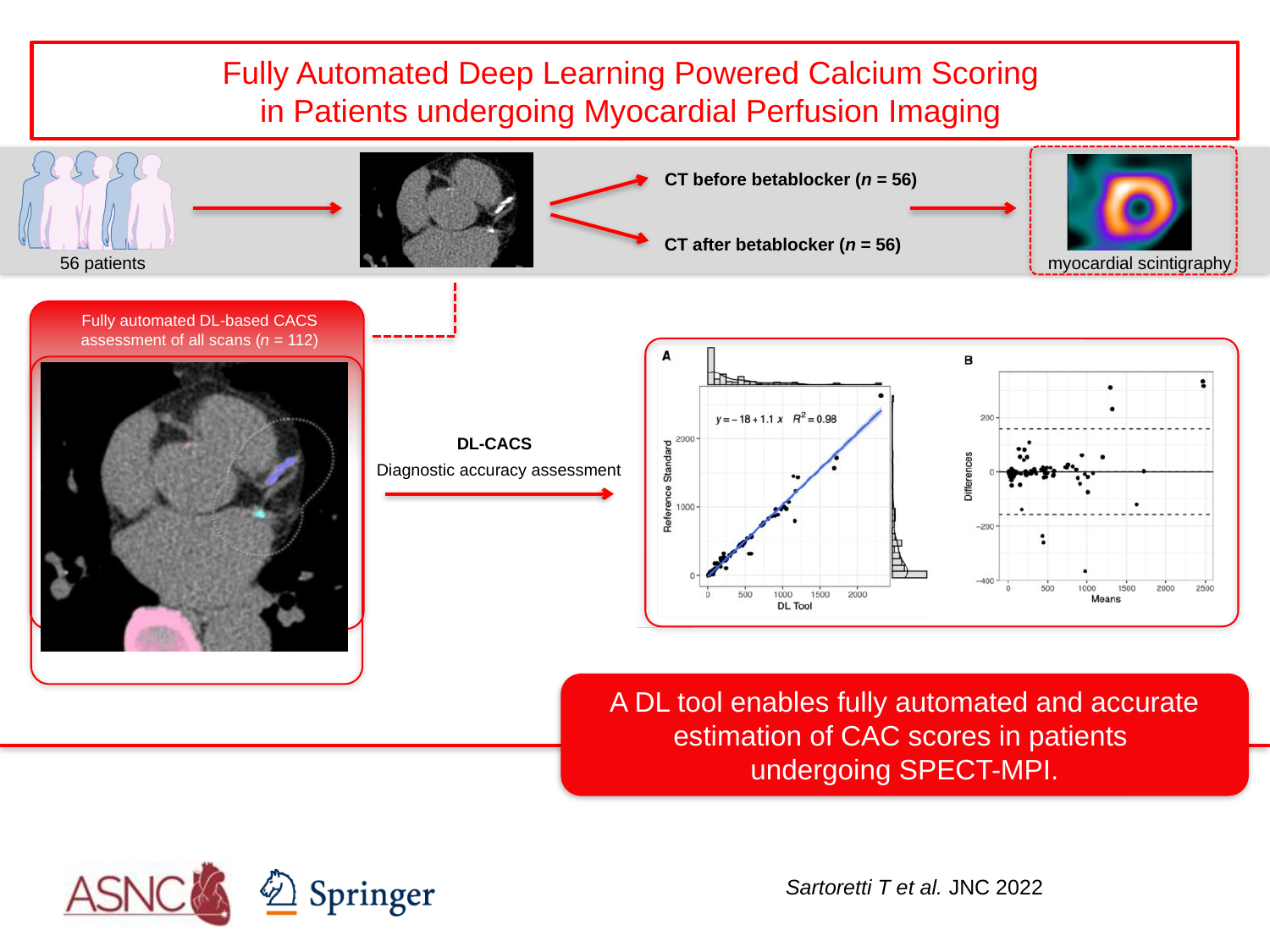

Fully Automated Deep Learning Powered Calcium Scoring
in Patients undergoing Myocardial Perfusion Imaging
CT before betablocker (n = 56)
CT after betablocker (n = 56)
myocardial scintigraphy
56 patients
Fully automated DL-based CACS assessment of all scans (n = 112)
DL-CACS
Diagnostic accuracy assessment
49.3 HU
44.9 HU
45.5 HU
A DL tool enables fully automated and accurate estimation of CAC scores in patients
undergoing SPECT-MPI.
Sartoretti T et al. JNC 2022
